# UNDERSTANDING THE SUBJECTIVITIES OF OLDER ADULTS WITH DEMENTIA IN A RESIDENTIAL CARE FACILITY

**DOI:** 10.1093/geroni/igae098.4354

**Published:** 2024-12-31

**Authors:** Lingyi Shen

**Affiliations:** Duke Kunshan University, Kunshan, Jiangsu, China (People’s Republic)

## Abstract

China has the largest population of people with dementia. However, inadequate care support is available, leading to families to being overwhelmed and compelled to send their loved ones to residential care facilities. While much attention has been paid to understanding institutional dementia care from the caregivers’ perspectives, seldom does research focus on the experiences of older adults with dementia, especially in China. This study aims to explore the subjectivities of older adults with dementia in a residential care facility in the Chinese context. Over a two-month period, participant observation was conducted with informal conversations in a dementia unit in eastern China. Based on the collective perception, reconstructed memory and oral histories, different individuals’ realities were articulated. Through verbal and physical behaviors such as resistance to care, othering and grouping, older adults with dementia constructed the understanding towards themselves and their surroundings, as well as their own meaning of life. Constant manifestation of intersubjectivity was also observed within the group. Despite their disrupted lived experiences, older adults with dementia maintained their established relationships, with family and beyond, and connections with the broader society through their performance of embodied experiences, disclosing the complexity of family dynamics in contemporary China and the social history of the country. The study also identifies elements that constrain their subjectivities, such as the overuse of antipsychotics and therapeutic lies. A new and dialectical perspective towards the relationship between older adults with dementia and the care institution is provided, informing the development of person-centered dementia care in China.

